# Supplementary material for: Neural mechanisms of parasite-induced summiting behavior in ‘zombie’ Drosophila
Source: eLife. 2023 May 15;12:e85410. doi: 10.7554/eLife.85410 (PMC10259475; doi:10.7554/eLife.85410)
Supplement: Supplementary file 1. — Genotypes are abbreviated for lines deposited at stock centers, for clarity. Stock centers are as follows: BDSC = Bloomington Drosophila Stock Center; KDSC = Kyoto Drosophila Stock Center; JRC = Janelia Research Campus. Functional/morphological annotations for the summit screen are abbreviated as follows: AM = AMMC; Ar = arousal; Ci = circadian; CX = central complex; Gr = gravitaxis; SO = subesophageal ganglion; MB = mushroom body; NM = neuromodulator & neurotransmitter; NP = neuropeptide; PI = pars intercerebralis. [file elife-85410-supp1.docx]

| **Genotype** | **Source** | **Screen annotation** |
| --- | --- | --- |
| *104y-Gal4* | BDSC:81014 | Gr:PI |
| *104y-Gal4*; *Cha-Gal80* | Derived from *104y-Gal4* & *Cha-Gal80* | PI |
| *acj6*^-^ | BDSC:30025 | Ar |
| *acj6-Gal4* | BDSC:30025 | Ar |
| *Akh^-^* | BDSC:84448 | Ar |
| *AstC^-^* | BDSC:84453 | NP |
| *c17-Gal4* | BDSC:39690 | Ar |
| *c41-Gal4* | BDSC:30834 | CX:Gr:SO:MB |
| *c708a-Gal4* | BDSC:50743 | AM:MB |
| *CCha1*^-^ | BDSC:84458 | NP |
| *CCKR-17D1*^-^ | BDSC:84462 | NP |
| *CCLKR-17D3*^-^ | BDSC:84463 | NP |
| *Clk*^ar^ | BDSC:24513 | Ci |
| *Clk^Jrk^* | BDSC:24515 | Ci |
| *Cl^out^* | BDSC:56754 | Ci |
| *Clk4.1-Gal4* | BDSC:36316 | Ci |
| *Clk4.5-Gal4* | BDSC:37526 | Ci |
| *Clk856-Gal4/CyO; MKRS/TM6B* | Daniel Cavanaugh [(Gummadova et al., 2009)](https://paperpile.com/c/yz38Jl/u2R3) | Ci |
| *CNMa^-^* | BDSC:84485 | NP |
| *CNMaR^-^* | BDSC:84486 | NP |
| *cry-Gal4.Z16* | BDSC:24514 | Ci |
| *cry-Gal4.Z24* | BDSC:24774 | Ci |
| *cry^02^* | BDSC:86267 | Ci |
| *cry^b^* | BDSC:80921 | Ci |
| *cyc^01^* | BDSC:80929 | Ci |
| *DAT^-^* | BDSC:25547 | NM |
| *Dh31^-^* | BDSC:84490 | Ci:NP |
| *Dh31^KG09001^* | BDSC:16474 | Ci:NP |
| *DH31R^-^* | BDSC:84491 | Ci:NP |
| *disco^1^* | BDSC:5682 | Ci:Gr |
| DNc01 | JRC:SS04161 | Ar:PI |
| DNc02 | JRC:SS02395 | Ar:PI |
| DNp01 | JRC:SS00726 | Ar |
| DNp01 | JRC:SS00727 | Ar |
| DNp01 | JRC:SS02299 | Ar |
| *Dsk^-^* | BDSC:84497 | NP |
| *forS* | BDSC:76120 | Ar |
| *fru-Gal4* | BDSC:30027 | AM:PI |
| *GH86-Gal4* | BDSC:36339 | AM:PI |
| *gl^60j^* | BDSC:509 | Ci |
| *GLSNP3375-Gal4* | KDSC:104479 | AM:SO |
| *Hug-Gal4* | BDSC:58769 | Ar:SO |
| *iav-Gal4* | BDSC:52273 | AM:Gr |
| *Ilp1-Gal4* | BDSC:66005 | PI |
| *Ilp2-Gal4* | BDSC:37516 | Ar:PI |
| *Ilp3-Gal4* | BDSC:52660 | PI |
| *Ilp5-Gal4* | BDSC:66008 | PI |
| *JO-ACE-Gal4* | KDSC:113902 | Gr |
| *JO-CE-Gal4* | KDSC:113878 | Gr |
| *JO15-Gal4* | BDSC:6753 | Gr |
| *Kurs58-Gal4* | BDSC:80985 | PI |
| *MB010B-Gal4* | JRC:MB010B | Ar:MB |
| *Mmp2NP0509-Gal4* | KDSC:103625 | AM:CX |
| *nan-Gal4* | BDSC:24903 | Gr |
| *nan^36a^* | Kristin Scott  [(Kim et al., 2003)](https://paperpile.com/c/yz38Jl/39bF) | Gr |
| *NPF^-^* | BDSC:84549 | NP |
| *Oamb^-^* | BDSC:22758 | NM |
| *OctBeta1R^-^* | BDSC:18589 | NM |
| *Octbeta2R^-^* | BDSC:18896 | NM |
| *OctBeta3R^-^* | BDSC:24819 | NM |
| *Pdf-Gal4* | BDSC:6899 | Ci |
| *Pdf-Gal80, cry24-Gal4* | BDSC:80940 | Ci |
| *Pdf^-^* | BDSC:84561 | Ci:NP |
| *Pdf^01^* | BDSC:26654 | Ci:NP |
| *PdfR-Gal4* | BDSC:68215 | Ci |
| *PdfR^-^* | BDSC:84705 | Ci:NP |
| *PdfR^-^; DH31R^-^* | Derived from BDSC:84705, BDSC:84491 | Ci:NP |
| *PdfR^5304^* | BDSC:33068 | Ci:NP |
| *per-Gal4* | BDSC:7127 | Ci:SO:PI |
| *per^01^* | BDSC:80928 | Ci |
| *per^30^* | BDSC:63136 | Ci |
| *per^S^* | BDSC:80919 | Ci |
| *ple-Gal4* | BDSC:8848 | CX:MB:NM |
| *Proc^c04750^* | BDSC:11587 | NP |
| *Proc^MI06590^* | BDSC:42407 | NP |
| *ProcR^MB00909^* | BDSC:22930 | NP |
| *R10F08-Gal4* | BDSC:48441 | PI |
| *R10H10-Gal4* | BDSC:48445 | PI |
| *R11B09-Gal4* | BDSC:48288 | AM:SO:PI |
| *R11C01-Gal4* | BDSC:49240 | SO:PI |
| *R14F05-Gal4* | BDSC:49257 | Gr |
| *R16C05-Gal4* | BDSC:48718 | Ci |
| *R18H11-Gal4* | BDSC:48832 | Ci |
| *R19B09-Gal4* | BDSC:48840 | SO:PI |
| *R19G10-Gal4* | BDSC:47887 | PI |
| *R20A02-Gal4* | BDSC:48870 | Ar:CX |
| *R20E05-Gal4* | BDSC:48898 | Gr |
| *R21H04-Gal4* | BDSC:48958 | AM:SO |
| *R23E10-Gal4* | BDSC:49032 | Ar:CX:SO |
| *R25G04-Gal4* | BDSC:49136 | AM:SO:PI |
| *R26D11-Gal4* | BDSC:49323 | SO:PI |
| *R27A05-Gal4* | BDSC:49208 | SO:MB:PI |
| *R30G08-Gal4* | BDSC:48101 | CX:Gr |
| *R32G08-Gal4* | BDSC:49729 | AM:SO:PI |
| *R32H03-Gal4* | BDSC:49733 | AM:PI |
| *R34C05-Gal4* | BDSC:49778 | CX |
| *R43D05-Gal4* | BDSC:41259 | Ci:SO |
| *R44B02-Gal4* | BDSC:50199 | Gr |
| *R45B03-Gal4* | BDSC:50221 | AM:PI |
| *R46E11-Gal4* | BDSC:50272 | PI |
| *R47A08-Gal4* | BDSC:50288 | CX:PI |
| *R50C11-Gal4* | BDSC:38742 | SO:PI |
| *R50H05-Gal4* | BDSC:38764 | NM |
| *R51H05-Gal4* | BDSC:41275 | Ci |
| *R54D11-Gal4* | BDSC:41279 | Ci:PI |
| *R57F07-Gal4* | BDSC:46389 | SO:PI |
| *R61G12-Gal4* | BDSC:41286 | Ci |
| *R64C04-Gal4* | BDSC:39296 | AM:PI |
| *R64C10-Gal4* | BDSC:39301 | Ci:CX |
| *R65C07-Gal4* | BDSC:39344 | Ar:CX |
| *R65C11-Gal4* | BDSC:39347 | CX:PI |
| *R66B05-Gal4* | BDSC:39389 | SO |
| *R70F10-Gal4* | BDSC:39545 | AM:CX:SO:MB:PI |
| *R70G01-Gal4* | BDSC:39546 | AM |
| *R78G02-Gal4* | BDSC:40010 | AM:Ci:SO |
| *R85A11-Gal4* | BDSC:40415 | Gr |
| *R86H08-Gal4* | BDSC:40471 | Gr |
| *R91A01-Gal4* | BDSC:40569 | CX:PI |
| *R95E11-Gal4* | BDSC:40711 | PI |
| RNAi-*acj6* | BDSC:29335 | Ar |
| RNAi-*Akh* | BDSC:27031 | Ar:NP |
| RNAi-*Cry* | BDSC:51033 | Ci |
| RNAi-*Crz* | BDSC:25999 | Ar:NP |
| RNAi-*Crz* | BDSC:26017 | Ar:NP |
| RNAi-*CrzR* | BDSC:42751 | Ar:NP |
| RNAi-*DAT* | BDSC:31256 | NM |
| RNAi-*DAT* | BDSC:50619 | NM |
| RNAi-*DDC* | BDSC:27030 | NM |
| RNAi-*DDC* | BDSC:51462 | NM |
| RNAi-*Dh31* | BDSC:41957 | Ci:NP |
| RNAi-*Dh44* | BDSC:25804 | NP |
| RNAi-*for* | BDSC:21592 | Ar |
| RNAi-*for* | BDSC:31698 | Ar |
| RNAi-*Lk* | BDSC:25936 | NP |
| RNAi-*LkR* | BDSC:25836 | NP |
| RNAi-*Nplp2* | BDSC:53967 | NP |
| RNAi-*Nplp2* | BDSC:54041 | NP |
| RNAi-*Oamb* | BDSC:31171 | NM |
| RNAi-*Oamb* | BDSC:31233 | NM |
| RNAi-*Oct-Tyr* | BDSC:28332 | NM |
| RNAi-*OctAlpha2R* | BDSC:50678 | NM |
| RNAi-*OctBeta1R* | BDSC:31106 | NM |
| RNAi-*OctBeta1R* | BDSC:31107 | NM |
| RNAi-*OctBeta1R* | BDSC:50701 | NM |
| RNAi-*OctBeta1R* | BDSC:58179 | NM |
| RNAi-*OctBeta2R* | BDSC:34673 | NM |
| RNAi-*OctBeta2R* | BDSC:50580 | NM |
| RNAi-*OctBeta3R* | BDSC:31108 | NM |
| RNAi-*Pdf* | BDSC:25802 | Ci:NP |
| RNAi-*ple* | BDSC:25796 | NM |
| RNAi-*ple* | BDSC:65875 | NM |
| RNAi-*ple* | BDSC:76062 | NM |
| RNAi-*ple* | BDSC:76069 | NM |
| RNAi-*ppk25* | BDSC:27088 | Gr |
| RNAi-*ProcR* | BDSC:29414 | Ar:NP |
| RNAi-*ProcR* | BDSC:29570 | Ar:NP |
| RNAi-*ptp69D* | BDSC:29462 | Ar |
| RNAi-*ShakB* | BDSC:27292 | Ar |
| RNAi-*SifA* | BDSC:29428 | NP |
| RNAi-*SifA* | BDSC:60484 | NP |
| RNAi-*Tbh* | BDSC:27667 | NM |
| RNAi-*Tbh* | BDSC:67968 | NM |
| RNAi-*Tdc2* | BDSC:25871 | NM |
| RNAi-*Tk* | BDSC:25800 | Ar:NP |
| RNAi-*TkR86C* | BDSC:31884 | Ar:NP |
| RNAi-*TkR99D* | BDSC:27513 | Ar:NP |
| RNAi-*trh* | BDSC:25842 | NM |
| RNAi-*tutl* | BDSC:54850 | Gr |
| RNAi-*TyrR* | BDSC:25857 | NM |
| RNAi-*TyrR* | BDSC:57296 | NM |
| RNAi-*TyrRII* | BDSC:27670 | NM |
| RNAi-*TyrRII* | BDSC:64964 | NM |
| *ry^506^* | BDSC:225 | Ci |
| *RyaR^-^* | BDSC:84571 | NP |
| *shakB-Gal4* | BDSC:51633 | Ar:SO |
| *SifA-Gal4* | BDSC:84690 | PI |
| *sNPF^-^* | BDSC:84574 | NP |
| *SS00078-Gal4* | JRC:SS00078 | CX |
| *SS00090-Gal4* | JRC:SS00090 | CX |
| *SS00097-Gal4* | JRC:SS00097 | CX |
| *SS00117-Gal4* | JRC:SS00117 | CX |
| *SS01566-Gal4* | JRC:SS01566 | CX |
| *SS02214-Gal4* | JRC:SS02214 | CX |
| *SS02216-Gal4* | JRC:SS02216 | CX |
| *SS02255-Gal4* | JRC:SS02255 | CX |
| *SS02391-Gal4* | JRC:SS02391 | CX |
| *SS27853-Gal4* | JRC:SS27853 | CX |
| *SS50464-Gal4* | JRC:SS50464 | CX |
| *SS52578-Gal4* | JRC:SS52578 | CX |
| *Tbh^-^* | BDSC:56660 | NM |
| *Tdc-Gal4* | BDSC:9313 | SO:NM |
| *tim-Gal4* | BDSC:80941 | Ci:SO |
| *Trh-Gal4* | BDSC:38388 | SO:NM |
| *Trh-Gal4* | BDSC:38389 | CX:MB:NM |
| *Trh^-^* | BDSC:10531 | NM |
| *tutl-Gal4* | BDSC:63344 | AM:PI |
| *tutl-Gal4/CyO;Cha-Gal80* | Derived from BDSC:63344 and *Cha-Gal80/TM3, Sb* | PI |
| *tutl^1^/CyO* | Kendal Broadie  [(Bodily et al., 2001)](https://paperpile.com/c/yz38Jl/Mj7u) | Gr |
| *TyrR^-^* | BDSC:27797 | NM |
| *TyrRII^-^* | BDSC:23837 | NM |
| *UAS-PdfRg/CyO; UAS-Cas9/TM6B* | Matthias Schlichting [(Schlichting et al., 2019)](https://paperpile.com/c/yz38Jl/U2gp) | Ci:NP |
| *VT002215-Gal4* | JRC:VT002215 | SO:PI |
| *VTDh44-Gal4/TM3, Sb* | VT039046  (via Daniel Cavanaugh) | Ar:PI |
