# Supplementary material for: Neural mechanisms of parasite-induced summiting behavior in ‘zombie’ Drosophila
Source: eLife. 2023 May 15;12:e85410. doi: 10.7554/eLife.85410 (PMC10259475; doi:10.7554/eLife.85410)
Supplement: Supplementary file 2. — Genotypes for deposited lines are abbreviated for clarity (i.e. interrupted alleles are designated [-], most y and w alleles have been omitted). Stock centers are as follows: BDSC = Bloomington Drosophila Stock Center; KDSC = Kyoto Drosophila Stock Center; JRC = Janelia Research Campus. [file elife-85410-supp2.docx]

| **Figure** | **Panels** | **Experimental flies** | **Source** | **Control genotype, if applicable** | **Derived from** |
| --- | --- | --- | --- | --- | --- |
| Figure 1 | D-J | Canton-S |  |  |  |
| Figure 2 | B-E | See Supplementary File 1 | See Supplementary File 1 | Canton-S (mutants);  *R57C10-Gal4*/+ (RNAi); *UAS-TNT-E*/+ (Gal4) | BDSC:39171 BDSC:28837 |
| Figure 2 | F | R57C10 > TRiP-DH31 = *R57C10-Gal4*/+; *UAS-TRiP-DH31*+ | BDSC:39171 BDSC:41957 | *R57C10-Gal4*/+; *TM3, Sb[1]*/+ | BDSC:39171 BDSC:41957 |
| Figure 2 | F | R57C10>TRiP-Cry = *R57C10-Gal4*/*UAS-TRiP-Cry* | BDSC:39171 BDSC:51033 | *R57C10-Gal4/CyO* | BDSC:39171 BDSC:51033 |
| Figure 2 | F | *Clk[out]* | BDSC:56754 | *Clk[out]/+* | BDSC:56754 Canton-S |
| Figure 2 | F | *Clk[ar]* | BDSC:24513 | *Clk[ar]/+* | BDSC:24513 Canton-S |
| Figure 2 | F | *Dh31[KG09001]* | BDSC:16474 | *Dh31[KG09001]/+* | BDSC:16474 Canton-S |
| Figure 2 | F | Dh31 RNAi = *R57C10-Gal4*/+; *UAS-TRiP-Dh31*/+ | BDSC:39171 BDSC:41957 | *R57C10-Gal4/+; TM3, Sb/+* | BDSC:39171 BDSC:41957 |
| Figure 2 | F | *Pdf[01]* | BDSC:26654 | *Pdf[01]/+* | BDSC:26654 Canton-S |
| Figure 2 | F | *Dh31[-]* | BDSC:84490 | *Dh31[-]/+* | BDSC:84490 Canton-S |
| Figure 2 | F | *Clk[Jrk]* | BDSC:24515 | *Clk[Jrk]/+* | BDSC:24515 Canton-S |
| Figure 2 | F | *CNMa[-]* | BDSC:84485 | *CNMa[-]/+* | BDSC:84485 Canton-S |
| Figure 2 | F | *per[S]* | BDSC:80919 | *per[S]/+* | BDSC:80919 Canton-S |
| Figure 2 | F | *ss[1] cry[b]* | BDSC:80921 | *ss[1] cry[b]/+* | BDSC:80921 Canton-S |
| Figure 2 | F | *Dh31R[-]* | BDSC:84491 | *Dh31R[-]/+* | BDSC:84491 Canton-S |
| Figure 2 | F | *PdfR[5304]* | BDSC:33068 | *PdfR[5304]/+* | BDSC:33068 Canton-S |
| Figure 2 | F | *CNMaR[-]* | BDSC:84486 | *CNMaR[-]/+* | BDSC:84486 Canton-S |
| Figure 2 | F | *PdfR[-]*; *Dh31R[-]* | BDSC:84705 BDSC:84491 | *Dh31R[-]* | BDSC:84491 |
| Figure 2 | F | *Pdf[-]* | BDSC:84561 | *Pdf[-]/+* | BDSC:84561 Canton-S |
| Figure 2 | F | PdfR CRISPR = *R57C10-Gal4*/*UAS-PdfRg*; *UAS-Cas9*/+ | BDSC:39171 *UAS-PdfRg*/*CyO*; *UAS-Cas9*/*TM6B* | *R57C10-Gal4*/*CyO*; *UAS-Cas9*/+ | BDSC:39171 *UAS-PdfRg*/*CyO; UAS-Cas9*/*TM6B* |
| Figure 2 | G | fru = *fru-Gal4*/*UAS-TNT-E* | BDSC:28837 BDSC:30027 | *UAS-TNT-E*/+ | BDSC:28837 BDSC:30027 |
| Figure 2 | G | 104y - Cha = *104y-Gal4*/*UAS-TNT-E*; *Cha-Gal80*/*+* | BDSC:28837 *104y-Gal4, Cha-Gal80* | *CyO*/*UAS-TNT-E*; *Cha-Gal80*/+ | BDSC:28837 *104y-Gal4, Cha-Gal80* |
| Figure 2 | G | R54D11 = UAS-TNT-E/+; R54D11-Gal4/+ | BDSC:28837 BDSC:41279 | *R54D11-Gal4*/+ | BDSC:41279 Canton-S |
| Figure 2 | G | R27A05 = UAS-TNT-E/+; R27A05-Gal4/+ | BDSC:28837 BDSC:49208 | *R27A05-Gal4*/+ | BDSC:49208 Canton-S |
| Figure 2 | G | R19G10 = *UAS-TNT-E/+; R19G10-Gal4/+* | BDSC:28837 BDSC:47887 | *UAS-TNT-E/+; TM3, Sb/+* | BDSC:28837 BDSC:47887 |
| Figure 2 | G | per = *per-Gal4/UAS-TNT-E* | BDSC:28837 BDSC:7127 | *per-Gal4/+* | BDSC:7127 Canton-S |
| Figure 2 | G | tutl = *tutl-Gal4/UAS-TNT-E* | BDSC:28837 BDSC:63344 | *CyO/UAS-TNT-E* | BDSC:28837 BDSC:63344 |
| Figure 2 | G | R11B09 = *UAS-TNT-E/+; R11B09-Gal4/+* | BDSC:28837 BDSC:48288 | *R11B09-Gal4/+* | BDSC:48288 Canton-S |
| Figure 2 | G | SifA = *SifA-Gal4/UAS-TNT-E* | BDSC:28837 BDSC:84690 | *CyO/UAS-TNT-E* | BDSC:28837 BDSC:84690 |
| Figure 2 | G | R18H11 = *UAS-TNT-E/Bl; R18H11-Gal4/+* | BDSC:28837 BDSC:48832 | *Bl/UAS-TNT-E; TM6B/+* | BDSC:28837 BDSC:48832 |
| Figure 2 | G | Clk4.1 = *UAS-TNT-E/sna[Sco]; Clk4.1-Gal4/+* | BDSC:28837 BDSC:36316 | *UAS-TNT-E/sna[Sco]; TM6B/+* | BDSC:28837 BDSC:36316 |
| Figure 2 | G | R26D11 = *UAS-TNT-E/+; R26D11-Gal4/+* | BDSC:28837 BDSC:49323 | *UAS-TNT-E/+; TM3, Sb/+* | BDSC:28837 BDSC:49323 |
| Figure 2 | G | cry24 - Pdf = *cry24-Gal4/ X; Pdf-Gal80/UAS-TNT-E* | BDSC:28837 BDSC:80940 | *cry24-Gal4/ X; CyO/UAS-TNT-E* | BDSC:28837 BDSC:80940 |
| Figure 2 | G | VTDh44 = *UAS-TNT-E/+; VTDh44/+* | BDSC:28837 VT039046 | *UAS-TNT-E/+; TM3, Sb/+* | BDSC:28837 VT039046 |
| Figure 2 | G | Kurs58 = *Kurs58-Gal4/UAS-TNT-E; Pdf[01]/+* | BDSC:28837 BDSC:80985 | *Kurs58-Gal4/+; Pdf[01]/+* | BDSC:80985 Canton-S |
| Figure 2 | G | tim = *UAS-TNT-E/tim-Gal4* | BDSC:28837 BDSC:80941 | *tim-Gal4/+* | BDSC:80941 Canton-S |
| Figure 2 | G | R51H05 = *UAS-TNT-E/+; R51H05-Gal4/+* | BDSC:28837 BDSC:41275 | *R51H05-Gal4/+* | BDSC:41275 Canton-S |
| Figure 2 | G | tutl - Cha = *tutl-Gal4/UAS-TNT-E; Cha-Gal80/+* | BDSC:28837 *tutl-Gal4/CyO; Cha-Gal80* | *CyO/UAS-TNT-E; Cha-Gal80/+* | BDSC:28837 *tutl-Gal4/CyO; Cha-Gal80* |
| Figure 2 | G | Ilp3 = *Ilp3-Gal4/UAS-TNT-E* | BDSC:28837 BDSC:52660 | *CyO/UAS-TNT-E* | BDSC:28837 BDSC:52660 |
| Figure 2 | G | Clk856 = *Clk856-Gal4/UAS-TNT-E* | BDSC:28837 *Clk856-Gal4/CyO* | *CyO/UAS-TNT-E* | BDSC:28837 *Clk856-Gal4/CyO* |
| Figure 2 | G | Ilp2 = *Ilp2-Gal4/UAS-TNT-E* | BDSC:28837 BDSC:37516 | *CyO/UAS-TNT-E* | BDSC:28837 BDSC:37516 |
| Figure 2 | G | GH86 = *GH86-Gal4/X; UAS-TNT-E/+* | BDSC:28837 BDSC:36339 | *X/Y; UAS-TNT-E/+* | BDSC:28837 BDSC:36339 |
| Figure 2 | G | Pdf = *Pdf-Gal4/X; UAS-TNT-E/+* | BDSC:28837 BDSC:6899 | *X/Y; UAS-TNT-E/+* | BDSC:28837 BDSC:6899 |
| Figure 2 | G | Clk4.5 = *UAS-TNT-E/sna[Sco]; Clk4.5-Gal4/+* | BDSC:28837 BDSC:37526 | *UAS-TNT-E/sna[Sco]; TM6B/+* | BDSC:28837 BDSC:37526 |
| Figure 2 | H | R19G10 > syteGFP, DenMark =*UAS-syteGFP, DenMark/+; R19G10-Gal4/+* | BDSC:33064 BDSC:47887 |  |  |
| Figure 2 | I | Clk4.1 > TrpA1 = *UAS-TrpA1/+; Clk4.1-Gal4/+* | BDSC:26263 BDSC:36316 | Sib ctrl = *UAS-TrpA1/+; TM6B/+* | BDSC:26263 BDSC:36316 |
| Figure 2 | J | R19G10 > TrpA1 = *UAS-TrpA1/+; R19G10-Gal4/+* | BDSC:26263 BDSC:47887 | Sib ctrl = *UAS-TrpA1/+; TM6B/+* | BDSC:26263 BDSC:47887 |
| Figure 2 | K | R19G10>CsChrimson = *UAS-CsChrimson/+; R19G10/+* | BDSC:55135 BDSC:47887 | R19G10>CsChrimson = *UAS-CsChrimson/+; R19G10/+* | BDSC:55135 BDSC:47887 |
| Figure 3 | A | Aug21>GFP = *C(1)Dxyfv(X^X)/Y; Aug21-Gal4, UAS-GFP/CyO* | *C(1)Dxyfv(X^X)/Y; Aug21-Gal4, UAS-GFP/CyO* |  |  |
| Figure 3 | C,D | *w/+; Aug21-Gal4, UAS-GFP/UAS-DTI; tub-Gal80(ts)/+* at 29C | BDSC:25039 *Bl/CyO; tub-Gal80(ts) C(1)Dxyfv(X^X)/Y; Aug21-Gal4, UAS-GFP/CyO* | Sibling control: *w/+; Aug21-Gal4, UAS-GFP/UAS-DTI; tub-Gal80(ts)/+* at 21C | BDSC:25039 *Bl/CyO; tub-Gal80(ts) C(1)Dxyfv(X^X)/Y; Aug21-Gal4, UAS-GFP/CyO* |
| Figure 3 | E | Canton-S |  |  |  |
| Figure 4 | A-G | Canton-S |  |  |  |
| Figure 5 | A-C | *His2Av-mRFP* | BDSC:23651 |  |  |
| Figure 5 | D-F | PI-CA > mcd8GFP = *R19G10-Gal4/UAS-mcd8GFP* | BDSC:32185 BDSC:47887 |  |  |
| Figure 5 | G | Canton-S |  |  |  |
| Figure 5 | H | Canton-S |  |  |  |
| Figure 5 | I | Aug21>GFP = *w; Aug21-Gal4, UAS-GFP/CyO* | *w; Aug21-Gal4, UAS-GFP/CyO* |  |  |
| Figure 6 | A-D | Canton-S |  |  |  |
| Figure 1-figure supplement 1 | A-C | CantonS |  |  |  |
| Figure 1-figure supplement 2 | A-M | Canton-S |  |  |  |
| Figure 2-figure supplement 1 | A | R19G10>TNT-E = *UAS-TNT-E/+; R19G10-Gal4/+* | BDSC:47887 BDSC:28837 | Sib ctrl = *UAS-TNT-E/+; TM3, Sb/+* | BDSC:28837 BDSC:47887 |
| Figure 2-figure supplement 1 | B | Clk4.1>TNT-E = *UAS-TNT-E/sna[Sco]; Clk4.1-Gal4/+* | BDSC:28837 BDSC:36316 | Sib ctrl = *UAS-TNT-E/sna[Sco]; TM6B/+* | BDSC:28837 BDSC:36316 |
| Figure 2-figure supplement 1 | C | R18H11>TNT-E = *UAS-TNT-E/Bl; R18H11-Gal4/+* | BDSC:28837 BDSC:48832 | Sib ctrl = *Bl/UAS-TNT-E; TM6B/+* | BDSC:28837 BDSC:48832 |
| Figure 2-figure supplement 1 | D | All Clock>Kir2.1 = *Clk856-Gal4/+; UAS-eGFP:Kir2.1.FRT.mCherry/TM6B* | *Clk856-Gal4/CyO; 911-QF, QUAS-FLP/TM6, Sb UAS-eGFP-Kir2.1.FRT.mCherry (III)* | All Clock>mCherry = *Clk856-Gal4/+; UAS-mCherry.FRT.eGFP:Kir2.1/TM6B* | *Clk856-Gal4/CyO; 911-QF, QUAS-FLP/TM6, Sb UAS-mCherry.FRT.eGFP-Kir2.1 (III)* |
| Figure 2-figure supplement 1 | D | R43D05>Kir2.1 = *R43D05-Gal4/UAS-Kir2.1* | BDSC:41259 *UAS-Kir2.1* | *UAS-Kir2.1* |  |
| Figure 2-figure supplement 1 | D | per-Gal4>Kir2.1 = *per-Gal4/+; UAS-Kir2.1/+* | BDSC:7127 *UAS-Kir2.1* | *UAS-Kir2.1* |  |
| Figure 2-figure supplement 1 | D | Clock-DN1p>Kir2.1 = *Clk856-Gal4/+; 911-QF, QUAS-FLP/UAS-eGFP:Kir2.1.FRT.mCherry* | *Clk856-Gal4/CyO; 911-QF, QUAS-FLP/TM6, Sb UAS-eGFP-Kir2.1.FRT.mCherry (III)* | All Clock>mCherry = *Clk856-Gal4/+; UAS-mCherry.FRT.eGFP:Kir2.1/TM6B* |  |
| Figure 2-figure supplement 1 | D | DN1p>Kir2.1 = *Clk856-Gal4/+; 911-QF, QUAS-FLP/UAS-mCherry.FRT.eGFP:Kir2.1* | *Clk856-Gal4/CyO; 911-QF, QUAS-FLP/TM6, Sb UAS-mCherry.FRT.eGFP-Kir2.1 (III)* | All Clock>mCherry = *Clk856-Gal4/+; UAS-mCherry.FRT.eGFP:Kir2.1/TM6B* | *Clk856-Gal4/CyO; 911-QF, QUAS-FLP/TM6, Sb UAS-mCherry.FRT.eGFP-Kir2.1 (III)* |
| Figure 2-figure supplement 1 | D | Clk4.1>TNT-G = *UAS-TNT-G/+; Clk4.1-Gal4/+* | BDSC:28838 BDSC:36316 | *UAS-TNT-G/+; TM6B/+* | BDSC:28838 BDSC:36316 |
| Figure 2-figure supplement 1 | D | Clk4.1>TNT-C = *TNT-C/X or Y; Clk4.1-Gal4/+* | BDSC:28996 BDSC:36316 | *UAS-TNT-C/X or Y; TM6B/+* | BDSC:28996 BDSC:36316 |
| Figure 2-figure supplement 1 | D | R61G12>Kir2.1 = *R61G12-Gal4/UAS-Kir2.1* | BDSC:41286 *UAS-Kir2.1* | *UAS-Kir2.1* | *UAS-Kir2.1* |
| Figure 2-figure supplement 1 | D | Pdf-Gal4>Kir2.1 = *Pdf-Gal4/X; UAS-Kir2.1* | BDSC:6899 *UAS-Kir2.1* | *X/Y; UAS-Kir2.1/+* | BDSC:6899 *UAS-Kir2.1* |
| Figure 2-figure supplement 1 | D | Pdf-Gal4>rpr = *Pdf-Gal4/X; UAS-rpr/+* | BDSC:6899 BDSC:5824 | *X/Y; UAS-rpr/+* | BDSC:6899 BDSC:5824 |
| Figure 2-figure supplement 1 | D | Pdf-Gal4>hid = *Pdf-Gal4/X; UAS-hid/+* | BDSC:6899 BDSC:65403 | *Pdf-Gal4/X; UAS-hid/CyO* | BDSC:6899 BDSC:65403 |
| Figure 2-figure supplement 1 | E | Pdf-Gal4>hid = *Pdf-Gal4/X; UAS-hid/+* | BDSC:6899 BDSC:65403 | Sibling control = *Pdf-Gal4/X; CyO/+* | BDSC:6899 BDSC:65403 |
| Figure 2-figure supplement 1 | F | VTDh44>TNT-E = *UAS-TNT-E/+; VTDh44-Gal4* | *VTDh44-Gal4/TM3, Sb* BDSC:28837 | *UAS-TNT-E/+; TM3, Sb/+* | *VTDh44-Gal4/TM3, Sb* BDSC:28837 |
| Figure 2-figure supplement 1 | F | Dh44 RNAi = *elav-Gal4/X; UAS-Dcr2/+; UAS-TRiP-Dh44/+* | BDSC:25804 BDSC:25750 | Canton-S |  |
| Figure 2-figure supplement 1 | F | Dh44 RNAi = *R57C10-Gal4/+; UAS-TRiP-Dh44/+* | BDSC:39171 BDSC:25804 | *R57C10-Gal4/+* | BDSC:39171 Canton-S |
| Figure 2-figure supplement 1 | F | Hug-Gal4>TNT-E = *UAS-TNT-E/+; Hug-Gal4/+* | BDSC:28837 BDSC:58769 | *UAS-TNT-E/+* | BDSC:28837 Canton-S |
| Figure 2-figure supplement 2 | A-B | Clk4.1>TrpA1 = *UAS-TrpA1/+; Clk4.1-Gal4/+* | BDSC:36316 BSDC:26263 | Sib ctrl = *UAS-TrpA1/+; TM6B/+* | BDSC:36316 BSDC:26263 |
| Figure 2-figure supplement 2 | C-D | R19G10>TrpA1 = *UAS-TrpA1/+; R19G10-Gal4/+* | BDSC:47887 BSDC:26263 | Sib ctrl = *UAS-TrpA1/+; TM6B/+* | BDSC:47887 BSDC:26263 |
| Figure 2-figure supplement 2 | E-G | R19G10>CsChrimson = *UAS-CsChrimson/+; R19G10-Gal4/+* | BDSC:47887 BDSC:55135 |  |  |
| Figure 3-figure supplement 1 | A | *Akh[-]* | BDSC:84448 | *Akh[-]/+* | BDSC:84448 Canton-S |
| Figure 3-figure supplement 2 | B,C | CA ablation (NiPP1) = *C(1)Dxyfv(X^X)/X; Aug21-Gal4, UAS-GFP/+; UAS-NiPP1/+* at 29C | BDSC:23711 *C(1)Dxyfv(X^X)/Y; Aug21-Gal4, UAS-GFP/CyO* | *C(1)Dxyfv(X^X)/X; Aug21-Gal4, UAS-GFP/+; TM6C/+* at 29C | BDSC:23711 *C(1)Dxyfv(X^X)/Y; Aug21-Gal4, UAS-GFP/CyO* |
| Figure 3-S3 | D | *Aug21-Gal4, UAS-GFP/UAS-DTI; tub-Gal80(ts*) at 29C | BDSC:25039 *Bl/CyO; tub-Gal80(ts) C(1)Dxyfv(X^X)/Y; Aug21-Gal4, UAS-GFP/CyO* | *Aug21-Gal4, UAS-GFP/CyO; tub-Gal80(ts)* at 29C | BDSC:25039 *Bl/CyO; tub-Gal80(ts) C(1)Dxyfv(X^X)/Y; Aug21-Gal4, UAS-GFP/CyO* |
| Figure 3-figure supplement 2 | B-I | Canton-S |  |  |  |
| Figure 4-figure supplement 1 | A-B | Canton-S |  |  |  |
| Figure 5-figure supplement 1 | A | Canton-S |  |  |  |
| Figure 5-figure supplement 1 | B | PI-CA>mcd8GFP = *R19G10-Gal4/UAS-mcd8GFP* | BDSC:32185 BDSC:47887 |  |  |
| Figure 5-figure supplement 1 | C-D | *His2Av-mRFP* | BDSC:23651 |  |  |
| Figure 5-figure supplement 1 | E-F | Aug21>GFP = *w; Aug21-Gal4, UAS-GFP/CyO* | *w; Aug21-Gal4, UAS-GFP/CyO* |  |  |
| Figure 6-figure supplement 1 |  | Canton-S |  |  |  |
| Figure 6-figure supplement 2 | A-E | Canton-S |  |  |  |
